# Preparation of Drosophila Polytene Chromosome Squashes for Antibody Labeling

**DOI:** 10.3791/1748

**Published:** 2010-02-09

**Authors:** Weili Cai, Ye Jin, Jack Girton, Jorgen Johansen, Kristen M. Johansen

**Affiliations:** Department of Biochemistry, Biophysics, and Molecular Biology, Iowa State University

## Abstract

Drosophila has long been a favorite model system for studying the relationship between chromatin structure and gene regulation due to the cytological advantages provided by the giant salivary gland polytene chromosomes of third instar larvae.  In this tissue the chromosomes undergo many rounds of replication in the absence of cell division giving rise to approximately 1000 copies.  The DNA remains aligned after each replicative cycle resulting in greatly enlarged chromosomes that provide a unique opportunity to correlate chromatin morphology with the localization of specific proteins.  Consequently, there has been a high level of interest in defining the epigenetic modifications present at different genes and at different stages of the transcription process. An important tool for such studies is the labeling of polytene chromosomes with antibodies to the enzyme, transcription factor, or histone modification of interest. This video protocol illustrates the squash technique used in the Johansen laboratory to prepare Drosophila polytene chromosomes for antibody labeling.

**Figure:**
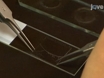


## Protocol

The following protocol for polytene chromosome squash preparation is adapted from the procedure described in Johansen *et al*. (2009).

### 1. Culture of third instar *Drosophila* larvae

In order to obtain optimal polytene chromosomes for high quality squash preparations, uncrowded culturing conditions are essential (i.e., place around 20 egg-laying female flies in a standard 4" fly bottle and change to a new bottle each day). Select the fattest individuals from the first crop of climbing 3rd instar larvae while they are still wandering but just prior to pupation. We routinely culture at 21°C but 18°C will yield fatter chromosomes that may be more suitable for some purposes such as for example when band/interband regions need to be visualized at high resolution.

### 2. Polytene squash materials

Drummond dissection forceps (2)
Petri dishes (60 x 15 mm)
Frosted microscope slides (Fisher No. 12-544-3) (poly-lysine-coated)
22 x 22 mm No. 15 cover slips (Fisher No. 12-520B) (coated with Sigmacote; Sigma #SL2)
22 x 40 mm No. 15 cover slips (Fisher No. 12-530B)
Kim-wipes
Phase contrast microscope with 20X objective
Small Dewar (e.g., vacuum flask or thermos bottle)
Long forceps
Razor blades
Coplin Jar
Rubber-Maid or Tupperware tray (or equivalent sealable tray)
Parafilm (cut into 22 mm squares)
175 g weights

### 3. Fixatives and solutions

5X formaldehyde stock. Prepare a fresh solution of 0.74 g. paraformaldehyde in 4.0 ml of dH_2_O and with 28 μl of 1N KOH. Warm to 65°C to dissolve the paraformaldehyde and then store on ice.
Fixative 1. Prepare a fresh solution of 0.5 ml 10X PBS, 50μl Triton X-100, 3.45 ml dH_2_O and 1.0 ml from a 5X formaldehyde stock. Warm to disperse the Triton X-100 and use within 1 hour.
Fixative 2. Prepare a fresh solution of 1.5 ml dH_2_O, 2.5 ml Glacial Acetic Acid, and 1.0 ml from a 5X formaldehyde stock and use within 1 hour.
Lactoacetic acid solution. Prepare a solution of 1 ml lactic acid, 2 ml dH_2_O, and 3 ml acetic acid.

### 4. Polytene chromosome squash preparation:

Rinse larvae with water and transfer to PBS in a tissue culture dish for dissection.
Grasp the tip of the mouth hooks with one pair of forceps, hold the body about 2/3 of the way down with the other pair, and pull on the mouth hooks so the salivary glands are exposed.  Separate the salivary glands from the brain and eye-antennal discs, and dissect away the fat body and any other associated tissues from the glands.
Add 200 microliters of Fixative 1 to the first well of a two well depression slide and 200-300 microliters of Fixative 2 to well two. Transfer one pair of salivary glands at a time to Fixative 1 in well one and incubate for the amount of time required for the target epitope, usually about 1-2 minutes. (Note: some epitopes, such as for most of the histone modifications, may require 5 minutes or longer fixation.)
Using forceps transfer the salivary glands to Fixative 2 in well 2 and incubate for two minutes.
Transfer the glands to 10-30 μl of Lactoacetic acid solution on a clean Sigmacoted coverslip. Gently lower a polylysine-coated microscope slide onto the coverslip and, without applying vertical pressure, pick up the coverslip so the glands are between the slide and the coverslip. *Immediately* facilitate cell lysis and chromosomal spreading by carefully grasping the coverslip with forceps on one edge and gently moving it slightly back and forth, trying to minimize any vertical pressure on the tissue. Alternatively use the eraser side of a pencil or even a fingertip to gently move the coverslip back and forth. Note that any delay in moving the coverslip back and forth is likely to diminish spreading of the chromosomal arms as they will tend to become more rigid upon exposure to the Lactoacetic acid solution. Clouding of the solution is often a good indication of cell separation. Gently tapping the coverslip obliquely (to avoid vertical pressure) with the eraser side of a pencil a few times may also assist in chromosomal spreading.
Immediately examine the tissue under a phase contrast microscope with a 20 or 40X objective to determine whether the chromosomes are well-spread.
When the chromosomal spreading is sufficient, set the slide with the coverslip side down on a stack of clean Kim-wipes. Place a second Kim-wipe on top and flatten chromosomes by placing your thumb over where the coverslip is positioned and pressing firmly, avoiding any horizontal movement of the coverslip that would shear the chromosomes.
Examine the slide again under the microscope to determine if the preparation is suitable for the intended purpose. If chromosomes have moved significantly upon squashing, use less volume of Lactoacetic acid solution in your subsequent preparations. Repeat steps 4.1-4.8 until a sufficient number of suitable slides have been obtained. Place 175 g weights upon the coverslip of the slides.
Fill a small Dewar (e.g., thermos bottle) with liquid nitrogen. Using a long forceps, dip slide into liquid N_2_ until the boiling stops, remove slide, and immediately use the edge of a clean razor blade at one corner to flip off the coverslip. If slide will be used immediately, place in a Coplin jar with PBS at 4°C. Otherwise collect the slide into a Coplin jar filled with 95% ethanol. Examine the coverslip once the frost has sublimed away to confirm that the tissue adhered to the slide and did not remain with the coverslip. Repeat with the remainder of the slides until all slides are stored in either PBS (for use within several hours) or ethanol (for longer term storage).

### Representative Results

The first critical step for obtaining high quality polytene squash preparation is to grow fat larvae with big salivary gland nuclei. The second is good technique with the spreading procedure, which may take some practice. One tip for improved spreading success is to find the minimal volume of lactoacetic acid solution during the squashing step. This will promote sufficient spreading of chromosomes without generating excessive streaming forces that can wash chromosome arms away. It is also worth noting that any delay in moving the coverslip back and forth will reduce spreading of the chromosomal arms as they become more rigid when exposed to the Lactoacetic acid solution.

If everything goes well, there should be numerous well spread polytene chromosomes. Figure 1 shows an example of such a preparation, double labeled with a marker for interband regions in green and with a dye that stains the banded regions in blue. If insufficient spreading is obtained, the chromosomes will look like little balls, as shown in Figure 2. On the other hand, if too much spreading has occurred, the chomosomes will be too thin and extended or in some cases fragmented into small pieces as shown in Figure 3.


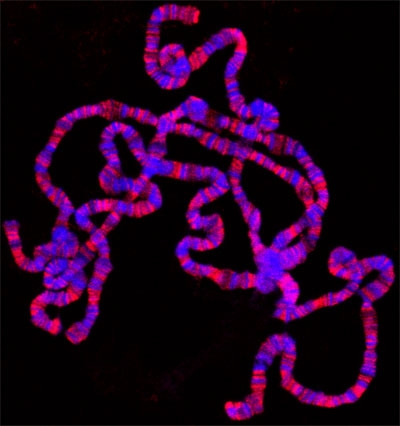
 **Figure 1.** Polytene squash preparation double labeled with antibody to the JIL-1 histone H3S10 kinase (in red) and Hoechst (blue).


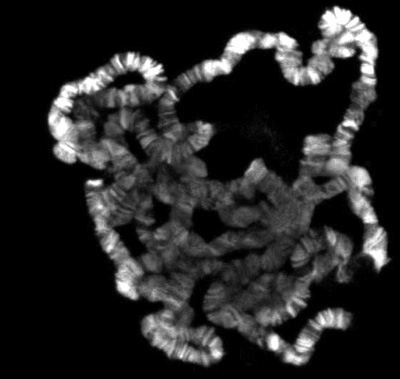
 **Figure 2.** Polytene squash with insufficient spreading.


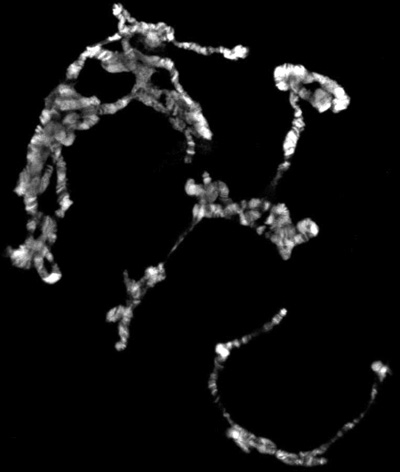
 **Figure 3.** Polytene squash with too much spreading.

## Discussion

The inclusion of acetic and lactic acids in conventional squash fixation protocols facilitates both interband resolution and chromosomal arm spreading but unfortunately some epitopes do not survive this treatment. An example of such an epitope is H3S10ph (Cai *et al*., 2008). Since acid treatment also has the disadvantage that it quenches the inherent fluorescence of GFP-tagged proteins, DiMario *et al*. (2006) recently developed a formaldehyde-based "acid-free squash technique" that allow for direct visualization of GFP-fusion protein on polytene chromosomes without GFP-antibody labeling as well as for antibody detection of acid-sensitive epitopes. The modifications for this procedure are further described in detail in Johansen *et al*. (2009). A representative acid fixed polytene squash preparation double labeled with antibody to the JIL-1 histone H3S10 kinase and Hoechst is shown in Fig. 1.
